# Cell wall integrity regulation across plant species

**DOI:** 10.1007/s11103-022-01284-7

**Published:** 2022-06-08

**Authors:** Luis Alonso Baez, Tereza Tichá, Thorsten Hamann

**Affiliations:** Institute for Biology, Faculty of Natural Sciences, Norwegian University of Science and Technology, 5 Høgskoleringen, 7491 Trondheim, Norway

**Keywords:** Cell wall integrity, Signaling, Environmental responses, Receptor-like kinases, Transcriptional regulation

## Abstract

Plant cell walls are highly dynamic and chemically complex structures surrounding all plant cells. They provide structural support, protection from both abiotic and biotic stress as well as ensure containment of turgor. Recently evidence has accumulated that a dedicated mechanism exists in plants, which is monitoring the functional integrity of cell walls and initiates adaptive responses to maintain integrity in case it is impaired during growth, development or exposure to biotic and abiotic stress. The available evidence indicates that detection of impairment involves mechano-perception, while reactive oxygen species and phytohormone-based signaling processes play key roles in translating signals generated and regulating adaptive responses. More recently it has also become obvious that the mechanisms mediating cell wall integrity maintenance and pattern triggered immunity are interacting with each other to modulate the adaptive responses to biotic stress and cell wall integrity impairment. Here we will review initially our current knowledge regarding the mode of action of the maintenance mechanism, discuss mechanisms mediating responses to biotic stresses and highlight how both mechanisms may modulate adaptive responses. This first part will be focused on *Arabidopsis thaliana* since most of the relevant knowledge derives from this model organism. We will then proceed to provide perspective to what extent the relevant molecular mechanisms are conserved in other plant species and close by discussing current knowledge of the transcriptional machinery responsible for controlling the adaptive responses using selected examples.

## Introduction

Cell walls are essential for plant development and tightly involved in many signaling processes. They provide mechanical support required for land plants growing on soil and are normally the first barriers involved in perception of stimuli and activation of signaling pathways inducing responses in the plasma membrane, cytoskeleton, cytoplasm, and organelles. Relevant stimuli during interactions between plants and their environment can have either a physical or chemical nature (Lamers et al. 2020; Le Gall et al. 2015). Physical stimuli include light, temperature, turgor pressure and mechanical forces, which affect the position of the cell wall with respect to the plasma membrane and could also include pressure in cytoskeleton structures (Bacete and Hamann 2020; Vaahtera et al. 2019). Chemical stimuli include non-plant derived molecules such as pathogen-associated molecular patterns (PAMPs) or plant-derived ones like damage-associated molecular patterns (DAMPs), volatiles, REACTIVE OXYGEN SPECIES (ROS) (including nitric oxide) and phytohormones (Chae et al. 2021; DeFalco and Zipfel 2021; Engelsdorf et al. 2018; Widhalm et al. 2015; Wong et al. 2021). Whether mechanical forces can also generate chemical signals by releasing components of the cell wall needs to be further explored.

Plants cells monitor the integrity of their cell walls and integrity impairment leads to responses mediated by the cell wall integrity (CWI) maintenance mechanism (Rui and Dinneny 2020; Somerville 2004; Vaahtera et al. 2019). CWI impairment is caused by cell wall damage (CWD) occurring during exposure to abiotic and biotic stress as well as growth and development. Receptor-like kinases and ion channels are typically involved in sensing CWI impairment and activating responses, including hormone biosynthesis and signaling, ROS, calcium transport and transcriptional regulation (Basu and Haswell 2020; Lamers et al. 2020; Chao Li et al. 2016a, b, c). Crosstalk between different pathways involving the same molecular components has made it difficult to fully comprehend the specific responses activated after exposure to a particular environmental stimulus or combination thereof. Nevertheless, CWI maintenance seems to be an attractive model system to study integration of signals induced by physical and chemical stimuli since previous work has shown that perception of both is relevant for integrity maintenance (Engelsdorf et al. 2018; Basu and Haswell 2020). Combining this model system with novel plant genome editing techniques that target specific genes and pathways should help us characterize gene hubs and genes with specific functions (Zhang et al. 2019; Zhu et al. 2020). The resulting knowledge will enable us to understand the mode of action of the mechanisms responsible for signal integration.

Typical methods to investigate CWI maintenance mechanisms involve the use of chemical agents or active enzymes to cause CWD (Engelsdorf et al. 2018). Cellulose is the main load-bearing component of cell walls and has been targeted by cellulose biosynthesis inhibitors (CBIs), like isoxaben (Desprez et al. 2002; Scheible et al. 2001). This pesticide affects the function of CELLULOSE SYNTHASE (CESA) proteins required for primary cell wall formation, resulting in reduced number of cellulose microfibrils leading to cell wall failure (Tateno et al. 2016). Interestingly, dicot plants are more sensitive to CBIs than monocots but the reason for this remains unknown (García-Angulo et al. 2012; Tateno et al. 2016). Techniques used to investigate cell wall composition and structure such as monoclonal antibodies, Fourier-transform infrared spectroscopy, Brillouin microscopy and labeling dyes are described in other reviews and will therefore not be reviewed here (Alonso-Simón et al. 2011; Prevedel et al. 2019; Rydahl et al. 2018; Ursache et al. 2021).

Most of the knowledge we currently have regarding the CWI maintenance mechanism and how it contributes to plant adaptation to environmental challenges comes from studies in *Arabidopsis thaliana* (hereafter Arabidopsis). Nevertheless, an increasing number of studies has started to shed light on the similarities and differences of CWI maintenance components in other plant species, including ferns, crops, and trees. Understanding how CWI functions in different plants species could have big economic impacts on plant-derived food and biofuel production (Ezquer et al. 2020; Ha et al. 2021). Climate change is causing extreme weather events that have profound negative impact on agricultural production (Intergovernmental Panel on Climate Change 2019). Therefore, understanding how plant cells react to abiotic and biotic stress will be beneficial for food crop productivity. In addition, plant biomass has the potential to be used as a sustainable resource to replace raw materials currently used for energy production or act as source for fine chemicals (Yoo et al. 2020). The latter is illustrated by lignin, which consists of monomers of interest to the chemical industry as precursors for plastics, and whose production is at least partially regulated by the CWI maintenance mechanism (Denness et al. 2011; Miedes et al. 2014). Therefore it is of particular interest to understand the CWI maintenance mechanism process as it can potentially enable us to become a more carbon–neutral society (European Commission. Directorate-General for Research and Innovation 2018).

Recent publications have focused on how mechanoperception affects CWI (Bacete and Hamann 2020), the relevance of differences in cell wall composition between monocots and dicots for CWI (Gigli-Bisceglia et al. 2020), and how these differences affect defense responses (Molina et al. 2021). Therefore, these topics will not be discussed here. In this review we will focus on chemical signals contributing to CWI maintenance and on the transcriptional regulation of relevant cell wall biosynthesis genes. We start by discussing the currently available information in Arabidopsis*,* proceed to summarize the knowledge available in other plant species and conclude by comparing and contrasting similarities and differences between molecular components and their interactions.

## Cell wall integrity maintenance: knowledge obtained from using *Arabidopsis* as model system

### The role of cell-surface receptors: CrRLK1L, WAK and their interactions

**Fig. 1:**
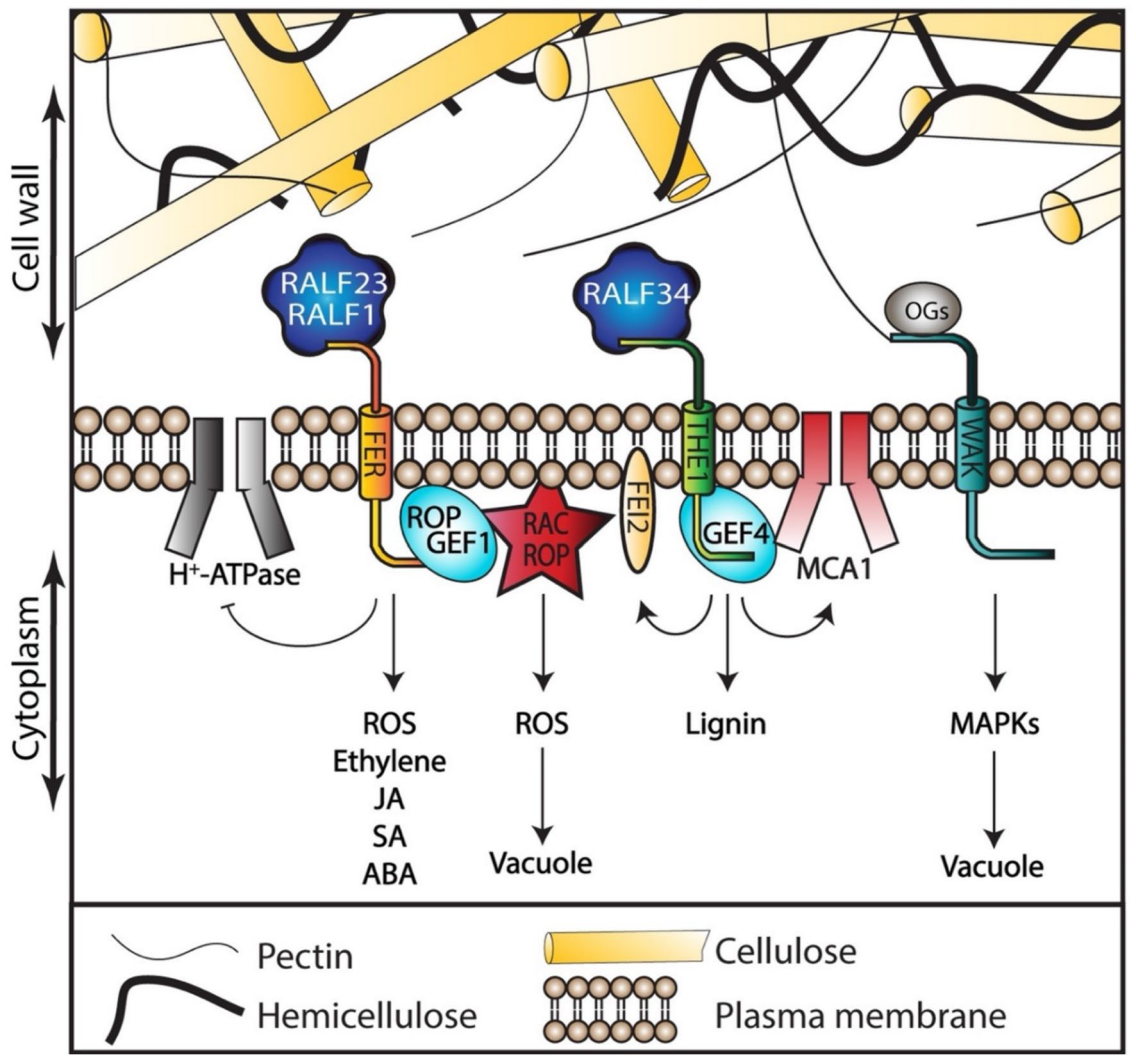
Receptors and their interactors participating in CWI maintenance in Arabidopsis. Membrane-bound receptor-like kinases can activate signaling pathways in response to environmental or chemical cues. Different interaction partners (co-receptors) and ligands (RALF peptides) seem to determine the specific activities of signaling process receptors, such as FER and THE1. Interaction with cell wall components (i.e. WAK or FER interactions with pectin) could trigger downstream signaling events

Environmental signals are mainly sensed in the cell wall-plasma membrane continuum (Fig. 1). Displacement between the two caused by mechanical forces can activate membrane-bound receptors and ion channels (Bacete and Hamann 2020; Basu and Haswell 2020; Codjoe et al. 2021; Yoshimura et al. 2021). Almost 20 different kinase protein families have been discovered in plants and several of them have been implicated in plasma membrane-located signaling processes (Dievart et al. 2020). Typically, the kinases contain a glycosylated extracellular ligand-binding domain, a hydrophobic transmembrane domain and a cytoplasmic kinase catalytic domain. Examples of such kinases are the members of the *Catharanthus roseus* receptor-like kinase1-like (*Cr*RLK1L) and Wall-associated kinase (WAK) subfamilies, which seem to interact with pectin via their extracellular domains (Decreux and Messiaen 2005; Feng et al. 2018; Gonneau et al. 2018; Kohorn 2016). This also supports the notion that they may function in sensing cell wall matrix disturbance. Different aspects of development, CWI maintenance, reproduction and response to stress are regulated by *Cr*RLK1L and WAK proteins but a large number of family members, their broad expression domains and the resulting redundancy has made it difficult to discern specific biological functions of individual family members (Franck et al. 2018).

The most studied member of this family, FERONIA (FER), illustrates this difficulty. FER has been implicated in many different biological processes including female fertility, cell elongation, immunity responses and mechanosensing (Höfte, 2015; Ortiz-Morea et al. 2021; Zhong et al. 2022). The role of FER in so many diverse processes has been explained by its capacity to bind to different interactors. Rapid alkalinization factor (RALF) peptides are growth regulators found in different plant species (Zhang et al. 2020b). FER seems to work as a scaffold, because it can interact with several different RALF peptides and other molecular interaction partners (Stegmann et al. 2017). FER functions as receptor for several RALFs including RALF1 (Abarca et al. 2021). The FER-RALF1 interaction enhances FER phosphorylation capacity and inhibits proton transport mediated by H^+^-ATPase pumps across the plasma membrane, which in turn probably affects modification of cell walls according to the acid growth hypothesis (Haruta et al. 2014). FER also regulates plant immunity by interacting with RALF23 to modulate elf18-induced ROS production (Xiao et al. 2019). Additionally, FER enhances flg22-induced FLS2-BAK1 complex formation, making FER an interactor/context-dependent positive or a negative regulator of plant immunity. This is illustrated by the phenotypes of *fer* loss of function mutants, which are hyposensitive to flg22, elf18 and chitin but more susceptible to *Pseudomonas syringae* pv. tomato DC3000 coronatine-minus strain (Stegmann et al. 2017). The FER-induced resistance is due to the activation of ethylene/jasmonic acid (JA) pathways (Kessler et al. 2010; Stegmann et al. 2017). FER also interacts with RHO OF PLANTS GUANINE EXCHANGE FACTOR 1 (ROPGEF1) and functions in the RAC/ROC (after the small family of GTPases Rho, Rac and Cdc42) signaling pathway to regulate ROS-mediated root hair growth (Duan et al. 2010) and to suppress abscisic acid (ABA) signaling by activating the phosphatase ABA INSENITIVE2 (ABI2) (Yu et al. 2012). FER also contributes to cell expansion by interaction with extracellular Leucine-rich repeat extensins (LRXs) to promote vacuole expansion (Dünser et al. 2019). The connection between the extracellular signal and vacuolar expansion has not been fully elucidated, but the current hypothesis suggests that actin dynamics, which are regulated by the FER-interacting RAC/ROP GTPases, could be modulating vacuole size. Actin dynamics have been recently shown to respond to osmotic stress (NaCl and mannitol) by changing the orientation of their filaments by 90 degrees in Arabidopsis and Chlamydomonas (Vilarrasa-Blasi et al. 2021). The FER-LRX-RALF module regulates the activity of salicylic acid (SA), JA, ABA and ROS pathways during plant growth under abiotic stress (Zhao et al. 2021). FER has also been implicated on the growth recovery phase after salt stress, where it is necessary to increase cytosolic calcium concentrations and to prevent cell bursting (Feng et al. 2018). This evidence suggests that FER could be implicated in vacuolar-driven cellular expansion upon exposure to abiotic stress, however the molecular mechanism remains to be determined.

THESEUS1 (THE1), the founder member of the *Cr*RLK1L gene subfamily, was identified as a suppressor of the short-hypocotyl phenotype observed in the cellulose-deficient *procuste1-1* (*pcr1-1*) mutant (Hématy et al. 2007). Knockout mutants (*the1-1*, *the1-2*, *the1-3* and *the1-6*) show no phenotypic defects in non-stressed conditions, suggesting that THE1 becomes active only if CWI has been impaired, supporting the notion that it functions as CWI sensor. The *the1-4* allele contains a T-DNA insertion in the region of the protein connecting the transmembrane and the kinase domains (Merz et al. 2017). Interestingly, in this allele, a truncated protein is produced that enhances the *prc1-1* phenotype, indicating that the kinase domain is not necessary for THE1 activation in cellulose deficient plants but for reducing its activity (Merz et al. 2017). Intriguingly the *the1-3* allele contains a T-DNA insertion 120 bases downstream from the *the1-4* insertion site and results in a loss-of-function allele (Merz et al. 2017). To explain the opposite effects, it was proposed that antisense transcripts (detected for both alleles but tenfold higher in *the1-3*) prevent gene expression in *the1-3* but not *the1-4*, thus making *the1-4* a gain-of-function allele. Furthermore, THE1 is required for cellulose-deficiency-induced lignin accumulation (Denness et al. 2011; Hématy et al. 2007). Importantly, THE1 does not influence cellulose deposition but rather senses cell wall perturbations (Hématy et al. 2007; Lindner et al. 2012). By combining a genetic analysis with measurements of JA, SA and lignin, it was shown that THE1 works in the same pathway as the mechanosensitive Ca^+^ channel MATING PHEROMONE INDUCED DEATH1 (MID1)—COMPLEMENTING ACTIVITY 1 (MCA1) and the RLK FEI2 regulating CWI (Engelsdorf et al. 2018). THE1 also interacts with a ROPGEF family member (ROPGEF4), similar to FER (Qu et al. 2017). This interaction seems to mediate plant defense responses against the necrotrophic fungi *Bortrytis cinerea*. THE1 functions as a receptor for RALF34 in a pH-dependent manner (Gonneau et al. 2018) to regulate lateral root initiation, with its specific mode of action to be determined.

Even though FER and THE1 have similar expression patterns in vegetative tissues and interaction partners from the same protein families (RALF and ROPGEF), their knockout phenotypes are drastically different. *the1* seedlings and plants only exhibit a visible phenotype when CWI is impaired (by treating them with a cellulose biosynthesis inhibitor or by combining them with cellulose-deficient mutants; Engelsdorf et al. 2018; Hématy et al. 2007). In contrast, *fer* plants exhibit very pronounced phenotypes, even in non-stressed conditions, exemplified by dwarf growth and reduced leave size due to reduced cell elongation (Deslauriers and Larsen 2010; Guo et al. 2009a, b). Mutations in other members of the *Cr*RLK1L family exhibit only minor effects on cell elongation (*HERCULES RECEPTOR KINASE1*; Guo et al. 2009a) and polar growth of root hairs (*ANXUR1* and *ANXUR2*;Boisson-Dernier et al. 2009; Miyazaki et al. 2009). This raises the intriguing question why mutations in only one family member (*FER*) result in diverse and often strong mutant phenotypes. This is particularly surprising since the majority of the family members have broad expression domains and their expression is activated by specific abiotic and biotic stresses (Franck et al. 2018; Lindner et al. 2012), implying that they could participate in responding to different environmental challenges. One possible explanation for the differential effects observed being that redundancy exists within the family, which does not include FER, leading then to the obvious question why FER is special.

As previously alluded to, FER plays essential roles in cell wall synthesis, stress response, cellular growth, morphogenesis and fertilization (Escobar-Restrepo et al. 2007; Franck et al. 2018; Guo et al. 2018; Shih et al. 2014). FER can bind to pectin fragments in vitro, to RALF peptides and also interact with LRXs. These characteristics have supported the notion that FER could act as a CWI sensor and core component of CWI maintenance (Feng et al. 2018; Shih et al. 2014). However, it is important to note that a *FER* knock down allele leads to enhanced production of JA, SA and lignin after exposure of *fer-5* seedlings to CWD, similar to the effects of the *the1-4* gain of function allele (Engelsdorf et al. 2018). These effects indicate that Arabidopsis plants can still perceive CWD (induced by cellulose deficiency) in the absence of FER. This suggests in turn that FER is not essentially required for detection of CWI impairment caused by cellulose biosynthesis inhibition, but is possibly required for perception of a different type of CWD or redundancy for certain FER activities does exist within the family.

The WAK subfamily contains 5 members in Arabidopsis, which are characterized by their conserved Epidermal growth factor (EGF)-containing extracellular domain, a transmembrane region and a conserved kinase domain (Kohorn 2016). Arabidopsis also encodes 21 WAK-like (WAKL) kinases, which show little similarity to the WAKs except in the EGF repeats and kinase domains, but it is not clear whether they associate with the cell wall (Verica and He 2002). WAK1, preferentially expressed in the vasculature, and WAK2, preferentially expressed in organ junctions, abscission zones and meristems, are the most abundant WAKs. WAK2 mutants exhibit shorter roots and reduction of vacuolar invertase levels, whereas the hyperactive receptor WAK2^cTAP^ induced stress responses, which were suppressed in a PECTIN-METHYLESTERASE3 (PME3) loss-of-function allele, suggesting that de-esterified pectin activates the WAK2 hyperactive allele. (Kohorn et al. 2012, 2009, 2006). This is in accordance with other data showing that WAKs can bind oligogalacturonides (OGs) and (preferentially) de-esterified pectin (Kohorn 2016). Other components of the signaling cascades in which WAKs are involved include MITOGEN-ACTIVATED PROTEIN KINASE 3 (MPK3), MPK6 and downstream targets of the signaling processes such as a vacuolar invertase, ENHANCED DISEASE SUSCEPTIBILITY 1 (EDS1) and PHYTOALEXIN DEFICIENT4 (PAD4), required for pathogen resistance as well as affecting turgor pressure (Kohorn 2016). While our knowledge regarding WAKs and WAKLs has improved significantly over recent years, we still do not fully understand their respective biological functions and mode of action nor identified their interaction partners. One possible way to resolve this situation could be to combine CRISPR-CAS-based manipulation of their respective activities with (phospho-)proteomics studies.

### Representative PAMPs and DAMPs relevant for CWI maintenance

**Fig. 2:**
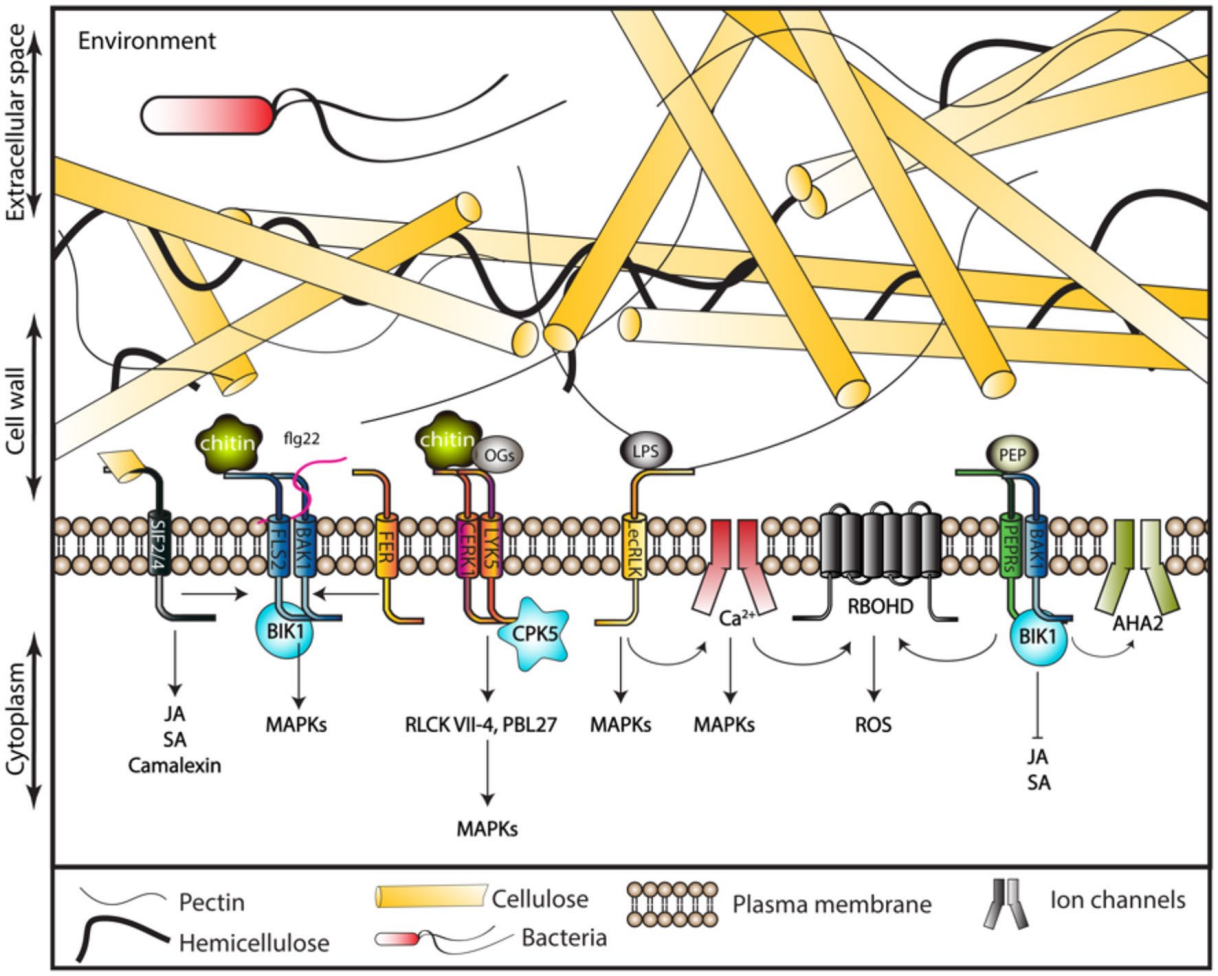
PAMPs and DAMPs activate diverse pathways in response to environmental stimuli. PAMPs and DAMPs are sensed at the plasma membrane by receptors from diverse families. Dependent on the specific perturbation and foreign or endogenous molecule detected specific defense responses are activated. Similar responses to biotic stress are observed in Arabidopsis and other plants, whereas chitin detection differs between Arabidopsis and rice (see text and Fig. 4)

As sessile organisms, plants have developed mechanisms to resist a variety of stresses. Pathogen infection can cause CWD during infection and activate the CWI maintenance mechanism (Hamann 2012; Novaković et al. 2018). Biotic stress responses are activated during plant infection by microbes and pathogens. Plant infection can occur through natural openings like stomata, open wounds, or direct infiltration by the usage of cell wall (CW)-degrading enzymes (Muthamilarasan and Prasad 2013). CW-degrading enzymes help to penetrate into the host tissues and make carbon sources and nutrients available to infecting pathogens (Cantu et al. 2008). Plants have evolved a multi-level protection system to prevent successful infection. The first level of protection consists of the physical-structural defense determined by cell wall composition and structure, which influence infection success or failure (Bacete et al. 2018). The second level is the response to signal molecules such as PAMPs and DAMPs. Plants can recognize specific invaders by their molecular patterns and activate specific defense mechanisms in response (Fig. 2; Abdul Malik et al. 2020; DeFalco and Zipfel, 2021; Zhou and Zhang 2020). DAMPs are produced by damaged cells or secreted from intact cells undergoing pathogen invasion. They typically consist of wall glycans, cytosolic proteins, protein fragments, peptides, nucleotides and amino acids (Hou et al. 2019). In contrast, PAMPs are secreted by invaders. PAMPs and DAMPs are recognized by pattern recognition receptors (PRRs), which are plasma membrane-localized receptor-like kinases (RLKs) or receptor-like proteins (RLPs) which activate plant immune responses (Li et al. 2020; Newman et al. 2013). The PRR family is divided into subfamilies according to their ability to perceive signals. Leucine-rich repeat receptor-like kinases (LRR-RLK), such as FLAGELLIN SENSING 2 (FLS2) and EF-TU RECEPTOR (EFR), perceive signals deriving from FLAGELLIN (flg22 epitope) and EF-Tu (elf18/elf26 epitopes). The PRR lysin-motif (LysM) RLK CHITIN ELICITOR RECEPTOR KINASE-1 (CERK1) binds to fungal chitin-oligomers and bacterial peptidoglycans (Couto and Zipfel 2016). P/DAMPs-triggered immunity (PTI) both involve a drastic increase in ROS production, activation of MAPK modules and Ca^2+^-DEPENDENT PROTEIN KINASES (CDPKs), modulation of hormone-based signaling, reorganization of the cytoskeleton and changes in gene expression (Ferrari et al. 2007; Giovannoni et al. 2021; Gravino et al. 2015; Kawasaki et al. 2017; Marti et al. 2021; Wong et al. 2007). These procedures also activate other defense mechanisms, which modulate CWI, cell wall composition/structure and help the plant to resist wall degradation. At the same time, activated signaling pathways also modify allocation of resources between growth and defense to maximise resources available to support defense responses (Lorrai and Ferrari 2021). CWI maintenance involves also accumulation of phytohormones such as JA, SA, and ethylene, increased production of ROS and activation of Ca^2+^-based and MAPK signaling modules. These inputs lead to ectopic production of cell wall components such as callose and lignin, originally implicated in defense responses (Denness et al. 2011; Ellis and Turner 2001; Engelsdorf et al. 2018; Hamann et al. 2009; Kohorn et al. 2009; Nakagawa et al. 2007; Tsang et al. 2011). Here we will focus on selected examples for PAMPs and DAMPs, because they can be used as tools to understand how CWI maintenance and PTI are controlling plant defense responses in a coordinated manner to successfully resist pathogen infection.

Flagellin is the main building block of the organs responsible for moving almost all flagellated bacteria and the primary ligand of receptors like FLS2 (Schuster and Khan 1994). Flagellin perception depends on FLS2 heterodimerization with the LRR-RLK co-receptor BRASSINOSTEROID INSENSITIVE 1-ASSOCIATED KINASE 1 (BAK1) followed by activation of signal translation elements such as BOTRYTIS-INDUCED KINASE 1 (BIK1) and MAPK species, PBS1-LIKE KINASES 1 (PBL1), and SUGAR TRANSPORT PROTEIN 13 (STP13), which leads to accumulation of hydrogen peroxide, reorganization of the cytoskeleton and increased hexose uptake (Henty-Ridilla et al. 2013; Lee and Seo 2021; Li and Staiger 2018; Macho and Zipfel 2014; Rasmussen et al. 2012; Zhang et al. 2010).

Plant defense responses induced by perception of chitin involve mainly secretion of chitinases (hydrolytic enzymes). However, a minor strategy involves recognition of fungal chitin by LYSIN MOTIF-CONTAINING RECEPTOR-LIKE KINASE 5 (LYK5) and CERK1 (Miya et al. 2007). LYK5 has a higher affinity to chitin than CERK1, which enhances the chitin-mediated formation of LYK5/CERK1 complex and CERK1 phosphorylation (Cao et al. 2014; Miya et al. 2007; Petutschnig et al. 2010). The signals generated by this complex are translated by RECEPTOR-LIKE CYTOPLASMIC KINASES (RLCK) VII-4 and PBL27, which in turn activate a MAPK signaling module (Bi et al. 2018; Rao et al. 2018; Yamada et al. 2016). CALCIUM-DEPENDENT PROTEIN KINASE 5 (CPK5) was recently implicated in the signaling process, because it can directly phosphorylate LYK5 and thus modulate chitin-induced defense responses in plants (Huang et al. 2020). Interestingly, chitin is not the only ligand that activates the CERK1/LYK5 complex. Callose-derived DAMPs, such as non-branched β-1,3-glucan oligosaccharides, promote immune responses in Arabidopsis through this complex as well (Mélida et al. 2018). Intriguingly, the juxta-membrane domains of CERK1, BAK1 and FLS2 all regulate the kinase activities of these receptors and play conserved roles in chitin signaling. This exemplifies the functionally similar organization of the receptor-based signaling processes mediating plant responses to chitin (Zhou et al. 2020).

Lipo-polysaccharides (LPS) form another relevant group of PAMPs (Kutschera et al. 2019; Shang-Guan et al. 2018; Sun et al. 2017). They act as ligands for the LECTIN RECEPTOR-LIKE KINASES (LecRLKs). LecRLKs bind non-enzymatically to specific carbohydrates, which function as links between the plasma membrane and cell wall (Gouget et al. 2006; Vaid et al. 2013). LecRLKs consist of an extracellular lectin domain, a transmembrane region and a cytoplasmic kinase region (Vaid et al. 2013). The classification of lectins is based on amino acid sequence, structure, as well as properties of the lectin domains and has led to a division of all lectins into three sub-classes. The first-class, C-type lectins require calcium ions for carbohydrate binding. The second-class, G-type lectins are *Galanthus nivalis* agglutinin-related lectins. The last-class are the L-type lectins, which were first discovered in Legume seeds (Bellande et al. 2017; Vaid et al. 2013). LPS can activate G-type PRR LIPOOLIGOSACCHARIDE-SPECIFIC REDUCED ELICITATION (LORE), which in turn phosphorylates the cytoplasmic receptor kinases PBL34, PBL35, and PBL36, leading to activation of a MAPK signaling pathway (Luo et al. 2020; Ranf et al. 2015; Sun et al. 2020). L-types LecRK-IX.2 and LecRK-I.9 (DOES NOT RESPOND TO NUCLEOTIDES 1 [DORN1]), which perceive flg22 and extracellular ATP, respectively; induce PTI responses including Ca^2+^ influx, direct phosphorylation and activation of RESPIRATORY BURST OXIDASE HOMOLOGS D (RBOHD) leading to ROS production and activation of the MAPK signaling module (Choi et al. 2014; Luo et al. 2017; Wang et al. 2018a, b).

PLANT ELICITOR PEPTIDES (PEPs) are well-established DAMPs and play an important role in PTI (Bartels and Boller 2015; Huffaker 2015; Lori et al. 2015). PEP precursors are sequestered at the vacuolar membrane. When cells are damaged, they are activated by METACASPASE4 (MC4) and released only in the damaged cells (Hander et al. 2019). PEPs bind to PEP-RECEPTOR1 and 2 (PEPR1 and PEPR2) and activate different responses. This includes interaction of PEPR2 with the proton pump AUTOINHIBITED H + -ATPase 2 (AHA2; Shen et al. 2020). AHA2 pumps protons into the apoplast, thus decreasing the pH in the apoplastic space (Shen et al. 2020). The PEP1/PEPR2 complex also interacts with BIK1 and RBOHD/F, activating a robust burst of ROS in response to bacterial pathogens (Jing et al. 2020). Intriguingly, application of PEPs suppresses in PEPR-dependent manner CWD-induced phytohormone production (Engelsdorf et al. 2018). Simultaneously, *PEPR1* and *PEPR2* loss of function mutants show enhanced JA, SA accumulation in response to CWI impairment (Engelsdorf et al. 2018). These results indicate that PTI and CWI maintenance-controlled processes are coordinated and PEP-based signaling processes are required for this coordination. One possible reason for this coordination could be that CWI maintenance acts as back-up defense mechanism activating phytohormone-based defenses in case the normal PTI-based responses are inhibited by pathogen-derived modulators.

DAMPs can also be cellulose-derived, since treatments with cellulose fragments (disaccharide to heptasaccharide) result in activation of PTI in a similar manner as OGs (Johnson et al. 2018; Souza et al. 2017). The most active compound is cellotriose, which induces ROS production, phosphorylation of MAPKs and expression of defense genes (Johnson et al. 2018; Locci et al. 2019). Such fragments could be generated by LYTIC POLYSACCHARIDE MONOOXYGENASES (LPMOs), enzymes used by phytopathogens to cleavage cellulose polymers (Vaaje-Kolstad et al. 2010). Two LRR-RLKs, namely STRESS INDUCED FACTOR 2 and 4 (SIF2 and SIF4), were implicated as critical factors in response to LPMO. LPMO treatments led to enhanced levels of ethylene, JA, SA, camalexin and increased resistance to the necrotrophic fungus *Botrytis cinerea* (Zarattini et al. 2021). In addition, physical interaction of SIF2 with the FLS2-BAK1 complex activates PTI signaling (Chan et al. 2020; Yuan et al. 2018). This results in direct phosphorylation of the SLOW ANION CHANNEL1 (SLAC1), which is necessary for abscisic acid (ABA)-mediated stomatal closure that enhances bacterial resistance (Chan et al. 2020).

One of the most studied groups of DAMPs are pectin-derived OGs, which activate PTI (Nothnagel et al. 1983) and were the first DAMPs discovered (Hahn et al. 1981). The core element of plant pectin is a polymer homogalacturonan (HG) that consists of α-1–4 linearly linked galacturonic acid molecules, which are methylesterified on C6 and often acetylesterified on C2 and/or C3 residues (Ridley et al. 2001). Production of OGs could be based on polygalacturonases that hydrolyze the α-1,4 linkages of low methyl-esterified homogalacturonans and have a substrate preference for linear polygalacturonic acids (Benedetti et al. 2015). However, a more recent report showed that treatment with *Botrytis cinerea* resulted in 80% of OGs being produced by pathogen-derived pectin lyases and acetyl- and methylesterified (Voxeur et al. 2019). Importantly, HG turnover also generates OGs, which could act as DAMPs starting immune responses or also have a signaling role during development (Denoux et al. 2008; Moscatiello et al. 2006). OGs promote expression of genes encoding chitinases, glucanases, and polygalacturonase-inhibiting proteins (De Lorenzo et al. 2001; Ridley et al. 2001); drastic increase of ROS production, accumulation of phytoalexins, activation of phospholipase C, CDPK and the cytoplasmic kinases ROG1 and ROG2 leading to increased expression of defense-related genes (Delteil et al. 2016; Ferrari et al. 2007; Galletti et al. 2008; Kohorn et al. 2016). The activity of OGs is dependent on the length, with a molecule size of 10–15 monomers apparently forming the most active forms (Ferrari et al. 2007). The pectin-hydrolyzing enzyme ARABIDOPSIS DEHISCENCE ZONE POLYGALACTURONASE 1 (ADPG1), which is required during development (Ogawa et al. 2009), has been reported recently to be upregulated in lignin-modified plants (Gallego-Giraldo et al. 2020). The changes in lignin composition apparently initiate a signaling cascade involving the CWI maintenance mechanism and induction of ADPG1 expression, releasing DAMPs (possibly OGs) and initiating immune responses (Voxeur and Höfte 2020). OGs also bind to WAK1 and 2, key regulators of cell expansion, biotic stress responses, wounding and metal tolerance (Kohorn et al. 2006; Tripathi et al. 2021). However, WAKs bind not only small pectin fragments but also long polymers cross-linked by Ca^2+^ in the cell wall and activate pectin-induced defense responses raising questions about specific functions of particular pectin types (Kohorn et al. 2009). To keep the deleterious effects of OGs under control, Arabidopsis encodes Berberine bridge enzyme-like (BBE-like) proteins whose oxidative activity specifically targets and inactivates the eliciting activity of non-oxidized OGs (Benedetti et al. 2018). Modification of OGs converts reducing galacturonic acids to oxidized galactaric acids. The change in homogalacturonan fragments retards degradation and use by microbial pathogens as carbon sources (Benedetti et al. 2018). It is unknown whether other molecules use similar oxidative mechanisms to regulate signaling and metabolic turn over of cell walls.

### Transcriptional regulation of cell wall metabolism

**Fig. 3:**
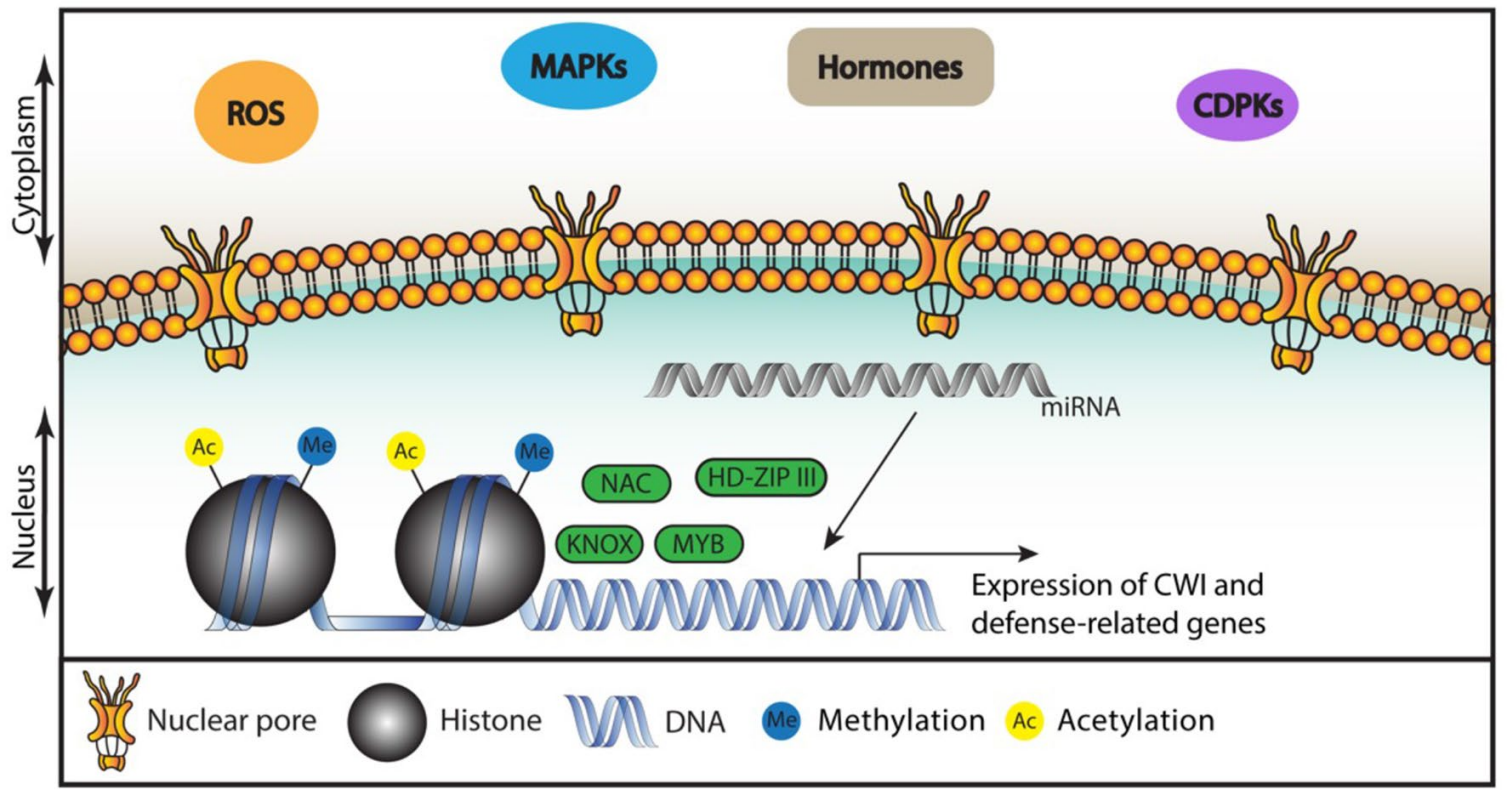
Transcriptional regulation of genes involved in responses to cell wall metabolism. An overview of relevant molecular components and structures in the cytoplasm and nucleus. Stress leads to generation of signals, which are relayed to the nucleus to modulate expression of transcriptional regulators, controlling expression of genes mediating responses to CWI impairment and biotic stress

Transcriptional regulation acting downstream of receptor-like kinases, small peptides or P/DAMPS has been linked mostly to immune responses. Examples are the interaction between FER and RALF peptides which lead to phosphorylation of the transcription factor (TF) MYC2 influencing JA signaling (Guo et al. 2018); the regulation of EDS1 and PAD4 by WAKs mediated by MAPKs mentioned above (Kohorn et al. 2016) and the activation of defense-related genes by P/DAMPS (B. Li et al. 2016a, b, c). The production of callose regulated by the TF MYB51 in response to flg22 illustrates how PTI regulates a particular element of cell wall metabolism (Clay et al. 2009). Many forms of CWD, both abiotic and biotic, activate common signaling processes involving hormone crosstalk, ROS and calcium signaling, meaning that the transcriptional machinery, controlled by these signaling processes, will be activated in response to CWD as well (Le Gall et al. 2015; Li et al. 2016a, b, c; Liao et al. 2017; Novaković et al. 2018). While CWI maintenance is likely to control transcriptional regulation of genes involved in cell wall metabolism, the mechanisms linking CWD perception to transcriptional changes remain to be elucidated. In addition to input from CWI monitoring, transcriptional regulation of cell wall biosynthesis genes needs to also integrate inputs deriving from perception of other stresses and developmental cues (Fig. 3). This ensures correct synthesis and delivery of cell wall components for specific cell types, developmental stages and during interactions with the environment (Kozlova et al. 2020; Rao and Dixon 2017; Wang et al. 2016; Wolf et al. 2012).

Little is known about transcriptional regulation during primary cell wall formation. In Arabidopsis, the Ethylene Response Factors (ERF) TFs *ERF034*, *ERF035*, *ERF038* and *ERF039* belong to group IIId of the ERF *APETALA2/ETHYLENE-RESPONSIVE ELEMENT BINDING PROTEIN* (AP2/EREBP) supergene family and function as transcriptional activators of *CESA1*, *CESA3* and *CESA6*, which are involved in primary (but not secondary) cell wall formation (Saelim et al. 2019). Ectopic expression of ERFs 035–038 and ERF040 (belonging to groups IIId and IIIe) in the double mutant *nac secondary wall thickening promoting factor 1 and 3* (*nst1and nst3*), lacking secondary cell walls, produced multilayered, thickened fiber cell walls without ultra violet autofluorescence indicative for the presence lignin, therefore resembling primary cell walls (Sakamoto et al. 2018).

Secondary cell wall regulation is better understood and involves coordination of multiple regulatory layers consisting of distinct classes of transcription factors (Kumar et al. 2016). Using high-spatial-resolution gene expression data and enhanced yeast one-hybrid assays, a gene regulatory network involving TFs and enzymes involved in secondary cell wall biosynthesis was mapped (Taylor-Teeples et al. 2015). E2 FACTOR C (E2Fc, from the E2F family of TFs, whose members are key regulators of the cyclinD/retinoblastoma/E2F pathway) seems to act upstream of the several TF families acting as secondary cell wall formation regulators: NAM (NO APICAL MERISTEM), ATAF (ARABIDOPSIS TRANSCRIPTION ACTIVATION FACTOR), CUC (CUP-SHAPED COTYLEDON) and VASCULAR-RELATED NAC DOMAIN 6 and 7 (VND6 and VND7) belonging to the NAC family of TFs. Moderate levels of E2FC activate VND7 whereas extremely low or high levels of E2FC repress *VND7* expression, in agreement with previous reports showing that E2FC can act as a transcriptional activator and repressor (de Jager et al. 2001; del Pozo et al. 2007; Heckmann et al. 2011; Kosugi and Ohashi, 2002). TFs belonging to this regulatory network were further classified in tiers, based on their regulatory hierarchy (Kumar et al. 2016). Tier 1 genes regulate transcription of structural genes, tier 2 regulate tier 1 and structural genes whereas tier 3 regulate tier 1 and 2 genes. VND6 and VND7 are positioned high in the hierarchy and together with NST1, NST2 and SECONDARY WALL-ASSOCIATED NAC DOMAIN PROTEIN 1 (SND1) form Tier 3. Tiers 1 and 2 contain TFs of the MYB TF family-like KNOTTED1-like homeobox (KNOX). TFs from the Homeodomain-leucine Zipper class III (HD-ZIP III) family are also positioned high in the regulatory hierarchy and are important for cell differentiation (Du and Wang, 2015). HD-ZIP III expression is regulated by microRNAs miR165/166, ensuring proper vasculature patterning (Fig. 3; Carlsbecker et al. 2010; Miyashima et al. 2011). These transcriptional networks regulate genes required for cell wall formation include cellulose biosynthesis genes such as *CESA4*, *CESA7* and *CESA8* or genes involved in hemicellulose and lignin biosynthesis. These selected examples provide some perspective on the complexity of the transcriptional networks responsible for regulation of cell wall metabolism and form the foundation to be used for understanding the mode of action of the corresponding processes in other plant species. More importantly, the examples also highlight our lack of knowledge regarding the mechanisms connecting initial perception of CWI impairment with transcriptional regulation of metabolic processes bringing about changes in structure and composition of cell walls, in particular primary cell walls.

### Epigenetic regulation of cell wall biosynthesis

Epigenetic control is important for cell fate maintenance by tissue-specific regulation of gene expression during differentiation processes (Lafos et al. 2011). Epigenetic modifications are induced by abiotic and biotic factors and can result in improved long-term adaptability of plants to unfavorable environmental conditions (Kumar et al. 2017). Epigenetic regulation in dicots and especially in *Arabidopsis thaliana* has been extensively studied in the context of development and stress responses (Liang et al. 2020; Pikaard and Mittelsten Scheid, 2014).

The acetyltransferase GENERAL CONTROL NONDEREPRESSIBLE 5 (GCN5) modulates directly expression of genes required for cell wall loosening such as *CHITINASE-LIKE 1* (*CTL1*), *POLYGALACTURONASE INVOLVED IN EXPANSION 3* (*PGX3*), and *MYB DOMAIN PROTEIN 54* (*MYB54*) in response to salt stress (Zheng et al. 2019). The importance is confirmed by *GCN5* knockout mutants resulting in dwarfed plants, deformed flowers, decreased root length, and reduced cellulose content (Hu et al. 2015; Zheng et al. 2019). GCN5 binds directly to sequences in the promoter of *CTL1*, which mediates interactions between cellulose microfibrils and hemicelluloses (Zheng et al. 2019). The H3K4-histone methyltransferase ARABIDOPSIS HOMOLOG of TRITHORAX1 (ATX1) acts as a positive regulator on the secondary cell wall formation during stem growth by activating expression of NAC TF family members SECONDARY WALL-ASSOCIATED NAC DOMAIN PROTEIN1 (SND1) and NAC SECONDARY WALL THICKENING PROMOTING FACTOR1 (NST1; Wang et al. 2021). *EXPANSIN* (*EXP*) genes play an essential role during fruit ripening and their expression is regulated by histone modifications such as H3K9/K14 acetylation and H3K27 tri-methylation (Mu et al. 2021). While the available evidence indicates that regulation of cell wall metabolism by epigenetic modification happens, neither are the underlying principals and regulatory mechanisms well understood, nor do we know to what extent this regulatory mechanism is relevant in the context of CWI-mediated changes in cell wall metabolism.

## Regulation of CWI maintenance in other plant species

### Receptor-like kinases: abundance and diversification in different plant species

**Fig. 4:**
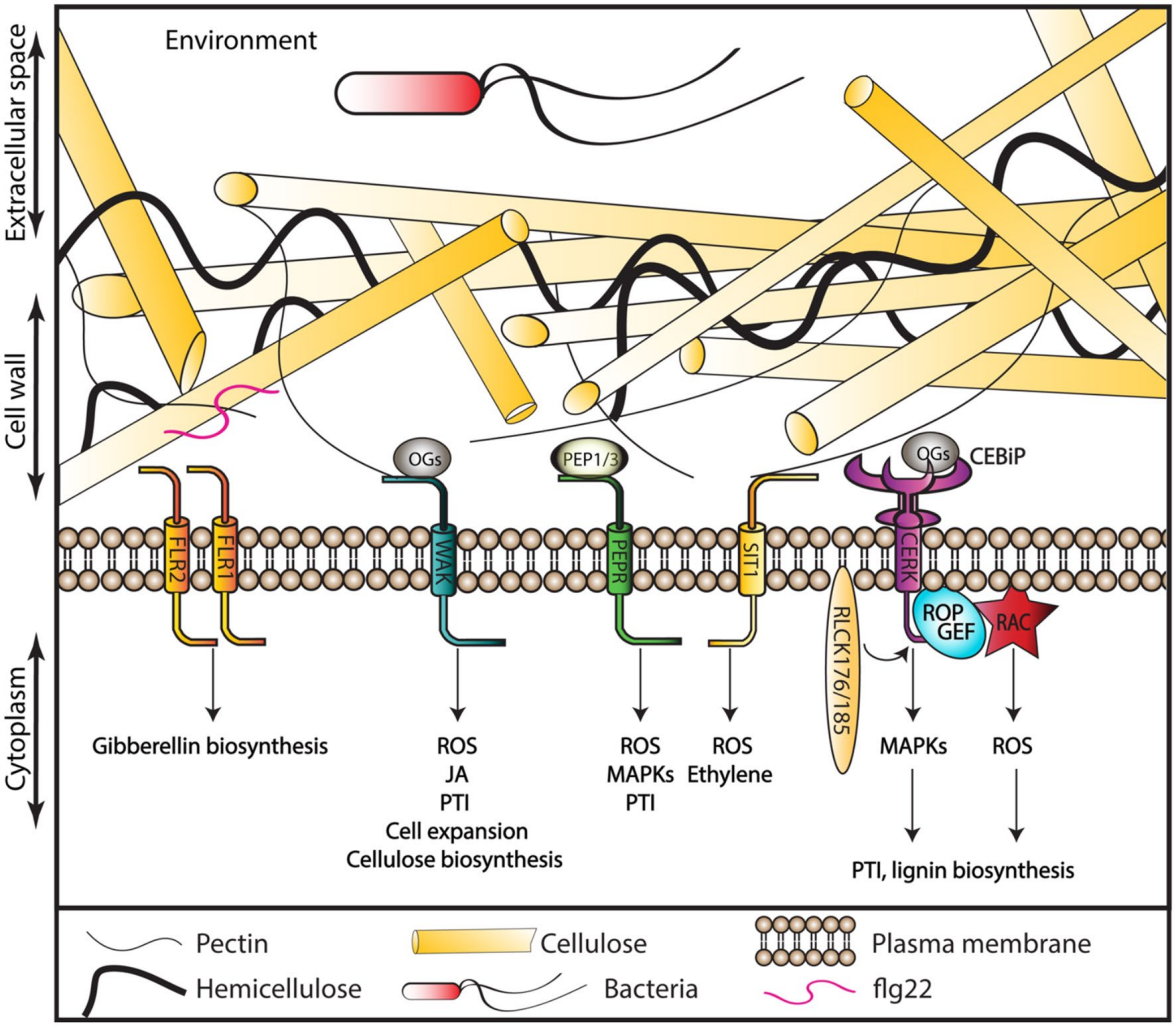
Receptors and biotic stress responses in other plants. Similar strategies to sense CWI impairment seem to be active in Arabidopsis and other plants. Since the number of family members encoding membrane-bound receptors and peptides has diversified in other plants, the specific interactions between receptors, co-receptors and ligands as well as the downstream effects of CWI impairment, can differ from the ones observed in Arabidopsis. FLR1 and FLR2 (*Oryza sativa*) are homologues of *At*FER with specialized functions in growth and immunity. WAKs family members have increased and specialized in crops including Brachypodium, *Zea maize*, wheat and *Oryza sativa*; where they function in diverse developmental and immune responses. Similar to their function in Arabidopsis, PEPs and PEPRs have been implicated in defense responses in *Zea maize* and *Oryza sativa*. SIT1 (L-type LecRLK) and CEBiP/CERK complex and interactors represented in the figure are based on *Oryza sativa* studies

Homologues of Arabidopsis RLK genes have been identified in other plant species (Couto and Zipfel, 2016; Honkanen et al. 2016), but conservation and diversification of their specific functions remain largely unexplored (Fig. 4). A recent study suggests that the *Cr*RLK1L kinase signaling pathway is specific for land plants, since analysis of algae genomes identified neither orthologues for *Cr*RLK1 members nor for RALFs peptides (Mecchia et al. 2020). The only *Cr*RLK1L kinase orthologue (*Mp*FER) found in *Marchantia polymorpha*, exhibits the highest similarity to FER from Arabidopsis, highlighting again its particular importance (despite having been referred to as *Mp*THE before) (Honkanen et al. 2016; Mecchia et al. 2020). *Mp*FER is required for correct rhizoid formation and expansion, maintaining the morphological integrity of the gametophyte and plant fertility, biological processes similar to those requiring *At*FER. However, *Mp*FER does not seem to repress cellular growth after CWI impairment, as *At*THE1. Isoxaben, a cellulose biosynthesis inhibitor, still affected the development of the gemmae in wild type and RNA interference lines targeting *Mp*FER (Mecchia et al. 2020), suggesting that the CWI maintenance function arose later during plant evolution.

The two main rice varietal groups *Oryza sativa indica* and *Oryza sativa japonica* contain the same number of *Cr*RLK1L kinases as Arabidopsis but very little information exists regarding their respective functions (Yang et al. 2020). FERONIA-LIKE RECEPTOR 2 and 11 (FLR2 and FLR11) seem to participate in plant immunity responses since *flr2* and *flr11* knock-out mutants exhibit enhanced resistance to rice blast infection without significant negative impact on growth. ROS production was increased at the penetration site after rice blast inoculation in leaf cells, while reduced ROS production and bigger lesion areas were observed in FRL2 overexpression lines (Yang et al. 2020). FRL2 seems to have a critical role in plant height, while FLR1 has a minor role but is required for fertility instead. FLR1 and FLR2 both positively regulate expression of genes encoding enzymes (ENT-KAURENE OXIDASE 2, GIBERELLIN 20-OXIDASE 2 and GIBERELLIN 30-OXIDASE 2) required for Gibberellin biosynthesis (Li et al. 2016a, b, c). Single mutants of the enzymes showed reduced shoot length, which was rescued to different extents by introducing *flr1* and *flr2*, suggesting that FLR1 and FLR2 are involved in different regulatory pathways.

A comprehensive genome-wide comparative analysis of the *Cr*RLK1L subfamily in 62 species found that on average eudicots contain 22 *Cr*RLK1L proteins whereas monocots only 13, possibly explained by the larger genome size of eudicots (Solis-Miranda et al. 2020). Additionally, a comparison between Arabidopsis and four legume species revealed that expression patterns of most *Cr*RLK1L genes were conserved despite the increased number of members in legumes (especially in the MEDOS clade). Based on the expression patterns of several legume *Cr*RLK1Ls they have been implicated in root nodulation.

The number of WAK kinases has increased profoundly in other plant species compared to Arabidopsis. Whereas this family consists only of 5 WAKs and 21 WAKLs in Arabidopsis (the distinction between WAKs and WAKLs is less clear in other species), the number has increased to 29 in cotton, 125 in rice, 341 in wheat, more than 100 in maize, 91 in Barley and 115 in *Brachypodium distachyon* (hereafter Brachypodium)(Dou et al. 2021; Tripathi et al. 2021; Wu et al. 2020; Zhang et al. 2021). Their ability to interact with pectin has been confirmed independently for several species, despite the low pectin abundance in grass cell walls (about 20% in primary cell walls of Arabidopsis but only 5–10% in grasses; Chen et al. 2021a, b; Gigli-Bisceglia et al. 2020; Wu et al. 2020) suggesting that WAKs have a highly conserved role in connecting pectic compounds in plant cell walls to intracellular responses. In Brachypodium, high expression levels of several WAK genes (specifically *BdWAK2, 10*, *42*, *72* and *108*) in rapidly growing tissue implicates them in cell expansion (Wu et al. 2020). Vacuolar invertase-dependent regulation of turgor pressure was hypothesized to be the mechanism responsible for WAK-mediated cell wall expansion in Arabidopsis but remains to be experimentally confirmed (Kohorn 2016; Kohorn et al. 2012). While WAKs have been implicated in abiotic and biotic stress responses by activating phytohormone biosynthesis, such as JA, and hypersensitive responses signaling cascades leading to programmed cell death, as exemplified by the activity of *Bd*WAK2 (Wu et al. 2020), the mechanisms responsible remain unknown. Interestingly, manipulation of wheat WAK and WAK-like genes confers pathogen resistance by different mechanisms. Wheat *WAK2* enhances resistance to the fungus *Fusarium graminearum* through interactions with pectin (Gadaleta et al. 2019). In contrast, gene-for-gene resistance to the fungus *Zymoseptoria tritici* producing Septoria tritici blotch (STB) is provided by *WAKL4*, also named *Stb6*, through recognition of apoplastic avirulent effectors (Saintenac et al. 2018).

### Relevant signaling processes during response to biotic stress

The mode of action of the signaling cascades activated by flagellin in Arabidopsis and rice exhibits similarities (Fig. 4). The *Cr*RLK1L FERONIA family in rice has 12 FERONIA-LIKE RECEPTORS (*Os*FLR) members (Yang et al. 2020). Overexpression of *Os*FLS2 results in enhanced immune responses to flg22 and flagellin (Takai et al. 2008). *Os*FLR1 has a crucial role in parasitism of *Meloidogyne incognita* where it interacts with parasite-derived RALFs and manipulates plant cell expansion and cell wall modification to facilitate parasitism (Zhang et al. 2020a). This is in contrast to the situation in *Triticum aestivum* (*Ta*), where transient silencing of *Ta*FER1 and *Ta*FER2 had no obvious impact on *Fusarium graminearum* infection (Wood et al. 2020).

In contrast, the mechanism modulating the response to chitin in rice differs from the one in Arabidopsis. Fungus derived chitin is recognized by LysM-RLP CHITIN OLIGOSACCHARIDE ELICITOR-BINDING PROTEIN (*Os*CEBiP), which afterward binds to (*Os*CERK1) whereas in Arabidopsis, *At*CERK1 binds chitin directly. The *Os*CEBiP/*Os*CERK1 complex in turn activates signaling components such as *Os*RacGEF (Akamatsu et al. 2013; Malinovsky et al. 2014; Shimizu et al. 2010). *Os*RAC1 is a small GTPase that activates immune responses such as ROS production by interacting with NADPH oxidase and a ROS scavenger to modulate the extent of ROS production (Wong et al. 2007). For proper response, both HEAT SHOCK PROTEIN 90 and its co-chaperone HSP70-HSP90 ORGANIZING PROTEIN (HOP) / STRESS-INDUCED PROTEIN 1 (STI1) are required because they are responsible for efficient transport of *Os*CERK1 and *Os*RAC1GEF from the endoplasmatic reticulum to the plasma membrane (Akamatsu et al. 2013; Chen et al. 2010; Nakashima et al. 2008). *Os*RAC1 is phosphorylated and activates then immune responses (including lignin biosynthesis involving CINNAMOYL-COA REDUCTASE 1 (*Os*CCR1)) through a MAPK-signaling module (Akamatsu et al. 2013; Kawasaki et al. 2017, 2006; Tang et al. 2017). RLPs like RECEPTOR-LIKE CYTOPLASMIC KINASE 185 (*Os*RLCK185) and RECEPTOR-LIKE CYTOPLASMIC KINASE 176 (*Os*RLCK176) are also positive regulators of immune responses in rice and interact directly with the *Os*CEBiP/*Os*CERK1 complex (Ao et al. 2014; Yamaguchi et al. 2019, 2013). Since PTI-controlled responses activated by chitin are quite pronounced, pathogens try to suppress them by secretion of LysM-containing proteins, which bind chitin oligomers creating complexes to avoid chitin recognition by the plant (de Jonge et al. 2010; Mentlak et al. 2012).

The LPS protein family has 75 members (32 G-type, 42 L-type, and 1 C-type) in Arabidopsis, and 173 members (100 G-type, 72 L-type, and 1 C-type) in rice (Vaid et al. 2012). In rice, ABNORMAL POLLEN 1 (AP1) belongs to the L-type LecRLKs and modulates carbohydrate metabolism required for pollen maturation (He et al. 2021). Another L-type LecRLK (SALT INTOLERANCE 1, SIT1) mediates salt-induced, ethylene-based signaling processes by modulating ethylene production and promoting ROS accumulation in rice (Li et al. 2014a, b). A G-type lecRLK is required for interactions between *Populus trichocarpa* and the ectomycorrhizal fungus *Laccaria bicolor* (Labbé et al. 2019). Simultaneously, it was shown that expression of LecRLK1 from *Populus trichocarpa* in non-host switchgrass roots allows colonization by *Laccaria bicolor* and improves productivity of this potential bioenergy crop exposed to abiotic stress (Qiao et al. 2021).

In *Zea mays, Zm*WAK genes play essential roles during infection by fungal pathogens *Sporisorium reilianum* and *Exserohilum turcicum* by regulating biosynthesis of defense-related benzoxazinoids (Hurni et al. 2015; Yang et al. 2019a, b; Zhang et al. 2017). Mutations in *Os*WAK genes (such as *Xa4*) lead to enhanced resistance against the bacterial blight (*Xanthomonas oryzae pv. oryzae-Xoo*), increased cell wall strength while reducing plant height to a small extent and not interfering with grain yield (Krattinger and Keller, 2017; Ning et al. 2017). In wheat, *Ta*WAK7D is modulating resistance against *Rhizoctonia cerealis*, by controlling expression of several pathogenesis-related genes (Qi et al. 2021). Comparative transcriptome analysis showed that the *Os*WAK activates TFs belonging to the bZIP, WRKY, MYB, DOF, and HSF families during bacterial infection, suggesting the TFs may be responsible for the enhanced resistance (Bakade et al. 2021).

DAMPs like the tri-saccharide 31-β-d-Cellobiosyl-glucose or the tetrasaccharide 31-β-d-Cellotriosyl-glucose arise when pathogen-derived endoglucanases digest rice hemicellulose. They also activate PTI in rice via *Os*CERK1 (Mélida et al. 2018; Yang et al. 2021). In rice, OGs do also activate WAK-mediated responses to biotic stress. These responses include enhanced cellulose biosynthesis (Hu et al. 2017), ROS production, and expression of genes involved in pathogen response (Delteil et al. 2016).

PEP-mediated processes have also been described in monocots (Lori et al. 2015). Infection with *Cochliobolus heterostrophus* and *Colletotrichum graminicola* activates *Zm*PEPR1, increases expression of defense-related genes and metabolites, while *Zm*PEP1 binds to *Zm*PEPR1 (Huffaker et al. 2011; Lori et al. 2015). Exogenous application of *Os*PEPs elicits multiple defense responses in rice cell cultures (Shinya et al. 2018). Treatment of rice cells with *Os*Pep3 during infection by *Mythimna loreyi* enhances defense responses, including activation of MAPKs and production of defense-related hormones and metabolites (Shinya et al. 2018). At the same time, *Os*PEPR1 overexpression increases the sensitivity of rice plants to these stress signals (Shinya et al. 2018).

### Transcriptional regulation of cell wall metabolism in different species

Our current knowledge regarding the regulation of primary cell wall formation in plants is based mainly on studies of Arabidopsis with the obvious consequence being that the process remains unexplored in many other species. Interestingly, coexpression experiments suggest that the rice homologue of *At*ERF34 is involved in secondary cell wall regulation, instead of primary cell wall regulation as in Arabidopsis (Hirano et al. 2013). Similar to Arabidopsis, transcriptional regulation of secondary cell wall formation in other plant species requires a diverse set of TF families including NAC, MYB, HD-ZIP III and KNOX (Kumar et al. 2016; Taylor-Teeples et al. 2015).

In rice, the KNOX TF *Os*KNAT7 coordinates secondary cell wall biosynthesis and cell expansion via different interactors and downstream factors compared to its Arabidopsis homologue, as also indicated by the differences in mutant phenotypes observed (Wang et al. 2019; Yu 2019a). Thicker walls in rice are caused by higher cellulose and xylan content, whereas in Arabidopsis this is caused by increased lignin. Additionally, rice mutants exhibit larger grain size and cells in spikelet bracts, effects not observed in Arabidopsis. Rice KNAT7 acts upstream of MYB61, thereby inhibiting secondary cell wall biosynthesis whereas in Arabidopsis KNAT7 is active downstream of MYB61 (Wang et al. 2019; Yu 2019b). It is unclear whether a feedback loop exists between both TFs or if secondary cell wall regulation evolved differently across species. *Os*KNAT7 interacts with GROWTH REGULATING FACTOR 4 (GRF4) and NAC31 to repress cell expansion and wall thickness. Furthermore, the cotton (*Gossypium hirsutum*) gene KNOTTED1-LIKE, member of the *Gh*KNOX family, regulates fiber development, initiation and elongation possibly by forming heterodimers with OVATE FAMILY PROTEIN 4 (Gong et al. 2014).

In Eucalyptus trees (*Eucalyptus grandis*), the NAC TF *Eg*NAC141 activates genes required for lignin biosynthesis (Sun et al. 2021). *Eg*NAC141 belongs to a group of genes identified through a combination of phylogenetics and large-scale expression profiling (Hussey et al. 2015). These genes are preferentially expressed in the xylem and have no Arabidopsis orthologues. NAC TFs involved in lignin production are characterized by their tissue- or cell-specific expression, as exemplified by *Eg*NAC141, which is apparently expressed 1000 times higher in the xylem and stem than in other tissues. Another NAC-domain containing protein, *Populus trichocarpa Pd*WND3A, which seems to be the homologue of the Arabidopsis *At*VND4 and 5 TFs, regulates vessel size in the stem (Yang et al. 2019a, b). Upregulation of ferulate 5-hydroxylase 1 gene expression in *Pd*WND3A overexpressing lines increases lignin content. The enzyme encoded mediates the chemical conversion from coniferaldehyde to 5-OH coniferaldehyde in the syringil monolignol biosynthesis pathway. It remains unclear if particular aspects of lignin biosynthesis are specifically regulated by *Pd*WND3A as both lignin biosynthesis and the ratio of lignin monomers syringil/guaiacyl are affected.

Expression of the TF HD-ZIP III family is regulated by microRNAs in Arabidopsis and rice (Carlsbecker et al. 2010; Miyashima et al. 2011; Zhang et al. 2018). *Os*miR166b is located in a yield-related QTL interval and affects rice grain yield (Fang et al. 2013). One of its targets, *Os*Hox32, regulates lignin and cellulose biosynthesis by suppressing expression of the cell wall biosynthetic genes CINNAMYL ALCOHOL DEHYDROGENASE 2 (*Os*CAD2) and *Os*CESA7 (H. Chen et al. 2021a, b). Knockdown lines of *Os*miR166b and overexpression of *Os*Hox32 exhibit culms with cavities, brittle culms and reduced cell wall thickness in leaves. These results suggest that *Os*miR166b and *Os*Hox32 form a regulatory module modulating culm formation. These selected examples illustrate how the functions of several TFs are conserved between different plant species, while others differ (Feller et al. 2011; Jiang, 2019; Wang et al. 2018a, b; Yokoyama and Nishitani, 2004). However, it is important to note that we observe both cases where individual genes have changed activities (ERF34) and where organisation of gene activities (KNAT7/MYB61) are modified.

### Epigenetic regulation of cell wall metabolism in crop plants

Epigenetic regulation in dicots and especially in *Arabidopsis thaliana* has been extensively studied in the context of development and stress responses (Yamaguchi 2019). In contrast, knowledge regarding epigenetic processes in monocots is limited. In protoplasts from Oryza, chromatin decondensation/reorganization and histone modification seem to be tightly connected to proteins required for de novo formation of cell walls (Mujahid et al. 2013; Tan et al. 2011). However, the mechanism regulating de novo wall formation in protoplasts differs probably from the normal situation, where the synthesis of new cell walls occurs during cell division, raising questions to what extent the regulatory mechanisms are the same (Mujahid et al. 2013). Salinity stress increased in *Zea mays* expression of histone acetyltransferase genes such as HISTONE ACETYLTRANSFERASES B (*Zm*HATB) and *Zm*GCN5*,* which can result in increased acetylation of H3K9 on at histone H4K5 (Li et al. 2014a, b)*.* The increased histone acetylation is associated with an enhanced expression of EXPANSIN B2 (*Zm*EXPB2) and XYLOGLUCAN ENDOTRANSGLYCOSYLASE (*Zm*XET1) genes (Li et al. 2014a, b; Wolny et al. 2021). These results are intriguing because salinity stress induces mainly elevated expression of *Zm*EXPB2, *Zm*EXPB6, and *Zm*EXPB8 isomers (Geilfus et al. 2010). In wheat, increased dimethylation at histone H3K9 and decreased levels of trimethylation at histone H3K4 and acetylation at histone H3K9 lead to a negative impact on transcriptional modulation of three EXPANSIN A1 (*Ta*EXPA1) homologs, gene silencing (Hu et al. 2013). Thus, monocots seem to sustain stable expression of β-Expansins in response to stress through epigenetic modification. In rice, GCN5 is highly expressed in the root meristem and is required for cell division and growth (Zhou et al. 2017). GCN5 is recruited by WUSCHEL (WUS)-RELATED HOMEOBOX11 (WOX11) and regulates root-specific genes involved in energy metabolism, hormone response and cell wall biosynthesis. To summarize the currently available information indicates that epigenetic modification is relevant for regulation of at least certain aspects of cell wall metabolism and that research opportunities exist to explore the mechanisms responsible.

## Conclusions

By monitoring the status of their cell walls, plants are able to adapt successfully to adverse environmental conditions. Sensing environmental stimuli at the wall triggers cellular responses responsible for successful adaptation to the surrounding environment. Progress has been made in Arabidopsis to identify the function of several CWI maintenance sensors and the processes they regulate. Nevertheless, the molecular mechanisms, interacting partners and regulatory pathways of most of the putative CWI sensors have still not been elucidated in detail. *At*FER function has been characterized extensively mainly because of the pleiotropic effects observed in the loss-of-function mutants, indicating that this protein has key functions in many different biological processes. This is to be expected bearing in mind the contributions of cell walls to many different aspects of plant life. *At*THE1 has also received attention because of its apparently more specific function in CWI maintenance in response to CWD. Interestingly, amongst higher plants, the *Cr*RLK1L gene family has fairly conserved numbers and expression domains for its members, suggesting that their function might also be conserved. In contrast, the number of WAK family members, which also seem to be able to detect cell wall impairment, is increased significantly in other plant species compared to Arabidopsis. However, WAKs and WAKLs continue to be involved in cell elongation and responses to biotic stress mechanisms in different plant species. Therefore, the reason for the increased number of genes and potential benefits of having them remains an open question. One possibility is that expression of several of these genes is restricted to specialized tissues and organs not found in other species.

Knowledge regarding the mode of action of the CWI maintenance mechanism and its relevance in other plant species is in its infancy. Larger genomes and multiple homologous genes, likely with redundant function, will pose difficulties in identifying the key molecular components for CWI maintenance. Investigating both ends of the evolutionary tree, close species to Arabidopsis on one side and ferns on the other, should help us discover common molecular mechanisms involved in the response to CWD and CWI maintenance and more importantly identify opportunities to use the resulting knowledge to improve performance of food and bioenergy crop plants.

In Arabidopsis, our knowledge regarding transcriptional regulation of primary cell wall metabolism has started to grow recently. This is exemplified by the ERF family of transcription factors, which regulate expression of CESA genes, thus controlling cellulose production. Regarding other metabolic processes required for primary cell wall formation, our understanding could be improved. However, comparing this knowledge to information regarding transcriptional regulation of the same metabolic processes in other species, highlights that the situation is there even worse, thus creating exciting research opportunities. In contrast, information, knowledge and understanding regarding transcriptional regulation of secondary cell wall metabolism in Arabidopsis and other plants is much more advance as exemplified by our knowledge regarding the networks of TFs from different families acting in a hierarchical manner to control secondary cell wall formation. For several Arabidopsis homologues the functions seem to be preserved in other species suggesting they could form leads to analyse the regulatory processes in these species in a knowledge-based manner.

## Data Availability

Not applicable.
